# Glutamate Delta 1 Receptor in Synapses, Circuits, and Disease

**DOI:** 10.1111/ejn.70415

**Published:** 2026-02-04

**Authors:** Diane Choi, Poojashree B. Chettiar, Yoland Smith, Shashank M. Dravid

**Affiliations:** ^1^ Graduate Program in Molecular and Systems Pharmacology Emory University Atlanta Georgia USA; ^2^ Department of Psychiatry and Behavioral Science Texas A&M University College Station Texas USA; ^3^ Department of Neurology Emory University Atlanta Georgia USA; ^4^ Emory National Primate Research Center Emory University Atlanta Georgia USA

**Keywords:** D‐serine, expression, GABA, GRID1, mutations

## Abstract

The glutamate delta 1 receptor (GluD1) remained largely unexplored since its cloning three decades ago because it lacked typical ligand‐gated ion channel activity. In the last decade, much progress has been made in identifying its potential function. This research has been greatly enhanced by the development of specific tools to determine receptor expression and distribution and genetic mouse models to explore region specific roles in regulating circuits and behavior. Major strides have also been taken in understanding the structure–function of the receptor. These studies demonstrate that GluD1 has many distinctive characteristics including synaptogenic activity at both excitatory and inhibitory synapses, the ability of the ligand‐binding domain to bind not only D‐serine but also GABA, its unique structural arrangement among the ionotropic glutamate receptor family in relation to domain swapping and the ability to induce tonic currents in the native system. Studies have also identified its role in regulating the postsynaptic content of AMPA and NMDA receptors and synaptic plasticity. Finally, human genetic studies revealed the relationship of GluD1 with neuropsychiatric disorders, including schizoaffective disorders and intellectual disability, which is consistent with the phenotypes observed in mice upon GluD1 ablation. The role of GluD1 is also becoming evident in neurological disorders, particularly chronic pain. Thus, GluD1 has quickly emerged as a receptor with multifaceted roles in physiology and pathology.

## Graphical abstract

This review discusses our current understanding of the structure–function, expression and roles in neural circuits of the less studied glutamate delta 1 (GluD1) receptor. Converging data suggests many unique features of GluD1 including expression at both excitatory and inhibitory synapses and binding to D‐serine and GABA. In addition, GluD1 has synaptogenic and synapse organizing properties that are critical for the regulation of circuits relevant to neuropsychiatric and neurological disorders.

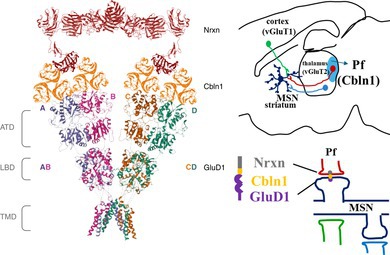

**Abbreviations**
ADHD: attention‐deficit/hyperactivity disorder
AMPA: α‐amino‐3‐hydroxy‐5‐methyl‐4‐isoxazolepropionic acid receptor
ASD: autism spectrum disorder
ATD: amino‐terminal domain
CTD: C‐terminal domain
EPSC: excitatory postsynaptic current
GABA: γ‐aminobutyric acid
GluA1–4: AMPA receptor subunits
GluD1/GluD2: Glutamate delta receptor 1/2
GluK1–5: kainate receptor subunits
GluN1/GluN2/GluN3: NMDA receptor subunits
GRID1: gene encoding the GluD1 receptor
ID: intellectual disability
iGluRs: ionotropic glutamate receptors
KO: knockout
LBD: ligand‐binding domain
mEPSC: miniature excitatory postsynaptic current
mIPSC: miniature inhibitory postsynaptic current
MSNs: medium spiny neurons
NMDA: N‐methyl‐D‐aspartate receptor
NMDAR: NMDA receptor
OCD: obsessive‐compulsive disorder
Pf: parafascicular nucleus
SZ: schizophrenia
TMD: transmembrane domain
VGAT: vesicular GABA transporter
vGluT1 /vGluT2: vesicular glutamate transporter 1/2
WT: wild type

## 1 Introduction

In the mammalian central nervous system, the bulk of excitatory synaptic transmission is mediated by ionotropic glutamate receptors (iGluRs). These transmembrane proteins are ligand‐gated ion channels, meaning that upon binding of a ligand, the receptor undergoes a conformational change to allow the influx of cations through the transmembrane pore. As their name suggests, iGluRs bind the neurotransmitter glutamate, which is released from the presynaptic terminal into the synaptic cleft. Subsequent activation of the postsynaptic iGluRs results in membrane depolarization, triggering an action potential. Given their widespread expression and distinct roles in mediating synaptic transmission, iGluRs dysfunction is implicated in a myriad of psychiatric and neurological disorders, thereby establishing them as important pharmacological targets.

**FIGURE 1:**
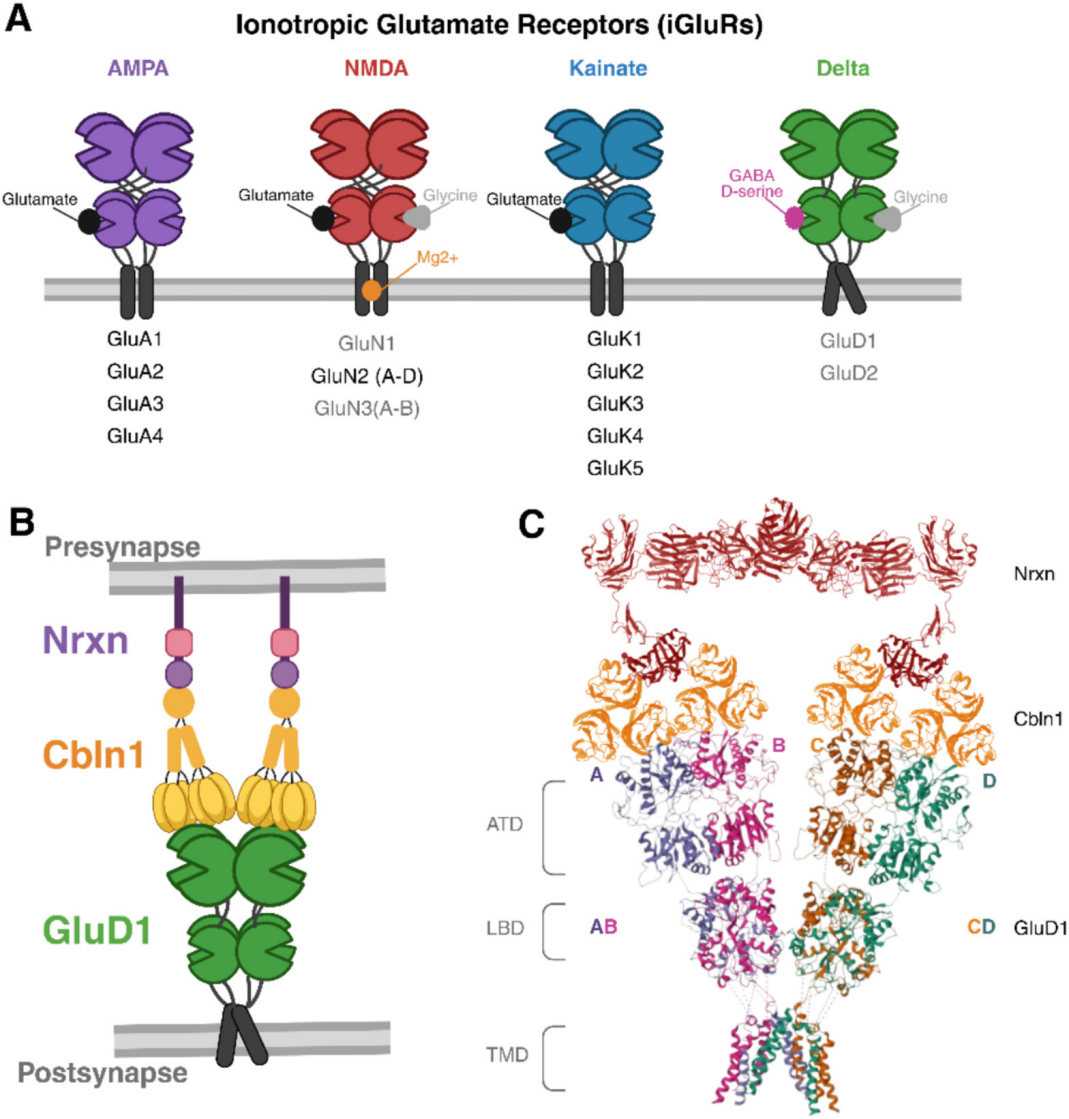
**Ionotropic glutamate receptor (iGluRs) subtypes and the structural assembly of the trans‐synaptic complex.** (A) Schematic representation of the four subfamilies of ionotropic glutamate receptors (iGluRs): AMPA (purple), NMDA (red), Kainate (blue), and Delta (green). AMPA and kainate receptors consist of GluA1–4 and GluK1–5 subunits, respectively, each containing a glutamate‐binding site (black). NMDA receptors are composed of GluN1, GluN2 (A–D), and GluN3 (A–B) subunits. GluN2 subunits bind glutamate (black), while GluN1 and GluN3 bind glycine (grey). Delta receptors include GluD1 and GluD2, which possess glycine‐binding sites but lack conventional ionotropic channel gating. (B) GluD1 (green) forms a tripartite bridge with cerebellin (yellow) and presynaptic neurexin (purple), facilitating synaptic organization and maintenance. (C) Structural organization of the GluD1–Cbln1–Neurexin trans‐synaptic complex, showing a high‐resolution model assembled from cryo‐EM and data. The postsynaptic GluD1 receptor (PDB: 6KSS) is shown at the bottom, adopting a nonswapped AB–CD domain architecture, where subunits A and B (purple, pink) and C and D (green, orange) pair within and across layers. The receptor comprises an amino‐terminal domain (ATD), ligand‐binding domain (LBD), and transmembrane domain (TMD), indicated at the left. Above GluD1 is the Cbln1 (PDB: 5H48; orange), forming a transsynaptic bridge that connects GluD1 to presynaptic Neurexin (PDB: 3POY; red), completing the tripartite complex.

The iGluRs superfamily consists of the N‐methyl‐D‐aspartate (NMDA), α‐amino‐3‐hydroxy‐5‐methyl‐4‐isoxazolepropionic acid (AMPA), kainate, and delta receptors (Figure 1). While the NMDA, AMPA, and kainate receptors are well studied for their roles in synaptic transmission and plasticity, the more recently discovered glutamate delta receptors (GluDs) are less understood. Identified in the early 1990s, GluDs are classified as iGluRs based on their sequence homology (~30%) with other iGluRs (Araki et al. 1993; Lomeli et al. 1993; Yamazaki et al. 1992). The two GluD subtypes, GluD1 and GluD2, share approximately 50% sequence homology (Lomeli et al. 1993). GluDs exhibit the same overall architecture as other iGluRs, comprised of four subunits that each consist of an extracellular amino‐terminal domain (ATD), a ligand‐binding domain (LBD), a transmembrane domain (TMD) that contains the ion channel pore, and a cytoplasmic C‐terminal domain (CTD) (Wo and Oswald 1995). However, GluDs are distinct in that they do not bind glutamate, nor do they display typical ligand‐induced ionotropic activity (Kristensen et al. 2016; Lomeli et al. 1993) (Figure 1). In the following sections on ligands, receptor interactions, and trans‐synaptic complex, we discuss both GluD1 and GluD2 since in many cases GluD2 has been the first to be examined.

## 2 Known Ligands of GluDs

GluDs were originally considered “orphan” receptors because they do not bind glutamate and have no known endogenous ligands (Araki et al. 1993; Lomeli et al. 1993). However, subsequent x‐ray crystal structure analyses of GluD2 LBD identified D‐serine and glycine as ligands that induce a closed‐cleft conformation in the LBD, similar to those observed in other iGluRs (Naur et al. 2007). Although these ligands did not produce typical ligand‐gated current in wildtype GluD2, additional studies involving the spontaneously open GluD2 lurcher mutant (GluD2^LC^), with A654T mutation in the highly conserved transmembrane region 3, have provided additional evidence (Wollmuth et al. 2000; Zuo et al. 1997). The binding of D‐serine and glycine, as well as extracellular calcium, inhibits the currents in GluD2^LC^ (Naur et al. 2007). Mutations to the same conserved transmembrane region 3 in GluD1 also result in constitutively open channels (Yadav et al. 2011). These studies demonstrated that GluDs do indeed have a functional channel pore, but they do not gate like other iGluRs. This may be due in part to the unique nonswapped architecture of their ATD and LBD, particularly for GluD1 (Burada et al. 2020a, 2020b) (Figure 1). GluD1 tetramer forms two‐fold symmetric dimers due to the lack of domain swapping at the NTD and LBD layer (Burada et al. 2020a; Kumar et al. 2023). Moreover, the lack of domain swapping also results in greater conformational flexibility of the extracellular domains, thereby destabilizing the dimer‐to‐dimer interaction of the NTD.

Building on these ligand studies, recently Piot et al. reported that GluD1 can also bind gamma‐aminobutyric acid (GABA) albeit with a very low affinity (Piot et al. 2023). GABA does not lead to ionic conductance in wildtype GluDs but in leaky GluD1^LC^ receptors (containing the A654T and C645I mutation), GABA, D‐serine, and glycine induced inward currents (Piot et al. 2023). GluD2^LC^ currents, which are inhibited by D‐serine and glycine (Naur et al. 2007), were however unresponsive to GABA, suggesting functional differences between the two GluD receptors. X‐ray crystallography studies of the GluD1 LBD bound to GABA or D‐serine further revealed a similar closed‐cleft conformation between the two ligands. Moreover, structure‐based mutagenesis experiments pointed to E446 as a key residue for GABA, but not D‐serine, binding in the LBD of GluD1 (Piot et al. 2023). Interestingly, a recent cryo‐EM study identified that GluD2 can bind not only D‐serine but also GABA and binding of these ligands induces conformational changes reminiscent of channel opening in other iGluRs (Wang et al. 2025). In relation to the native role of GABA binding to GluD1, ambient extrasynaptic GABA levels are in the low nanomolar range at rest (Farrant and Nusser 2005; Glykys and Mody 2007; Santhakumar et al. 2013), which increase during neurotransmission to reach ~1.5–3 mM (Jones and Westbrook 1995; Overstreet and Westbrook 2003). Because GluD1 binds GABA with a KD of ~2 mM and an EC_50_ of 3–12 mM (Masternak et al. 2023), it suggests that GluD1 is likely engaged by GABA during high‐frequency inhibitory activity.

## 3 Interactions Between GluDs and Other Receptor Subtypes for Ionic Function

Intriguingly, studies have demonstrated functional coupling between GluDs and other receptor subtypes, such as Gαq‐protein coupled receptors (GPCRs) (Ady et al. 2014; Benamer et al. 2018; Copeland et al. 2023; Dadak et al. 2016; Gantz et al. 2020). Specifically, multiple studies indicated that metabotropic glutamate receptor (mGluR) 1/5 activation can trigger the opening of GluD1 and GluD2 (Ady et al. 2014; Benamer et al. 2018; Dadak et al. 2016). Slow inward currents induced by the mGluR1/5 agonist 3,5‐dihydroxyphenylglycine (DHPG) were observed upon co‐expression of mGluR1 and GluD2 in HEK293 and cerebellar Purkinje cells (Ady et al. 2014; Dadak et al. 2016; Lemoine et al. 2020). mGluR1/5 activation was also shown to trigger the opening of GluD1 in HEK293 cells (Benamer et al. 2018). Moreover, GluD1 and GluD2 were shown to contribute to mGluR1/5‐dependent slow excitatory postsynaptic currents (EPSCs) in midbrain dopamine neurons (Benamer et al. 2018) and Purkinje cells (Ady et al. 2014), respectively. Gantz et al. further demonstrated a role for GluD1 in the mediation of α_1_‐adrenergic receptor‐dependent EPSCs in dorsal raphe neurons (Gantz et al. 2020; Khamma et al. 2022), expanding the repertoire of receptor systems that can gate GluD function. In parallel, several studies showed that GluDs carry a tonic current (Copeland et al. 2023; Gantz et al. 2020; Lemoine et al. 2020) that can be amplified by α_1_‐adrenergic receptors or by a mechanism independent from GPCR activity (Copeland et al. 2023; Gantz et al. 2020). Together these findings suggest that GluD1 may interact with GPCR to generate ionic conductance. It remains to be tested whether this form of ion channel activity is ligand‐dependent or independent.

## 4 The Trans‐Synaptic Complex

Studies over the last decade have identified that GluDs function as synaptic organizers, forming a trans‐synaptic complex with the presynaptic protein neurexin (Nxn) via cerebellin (Cbln) secreted in presynaptic terminals to regulate synapse formation and maintenance (Elegheert et al. 2016; Matsuda et al. 2010; Uemura et al. 2010). Nxns are presynaptic adhesion molecules, well‐studied for their various roles in synaptic transmission in different neuronal populations (see Review (Südhof 2017)). Their structure consists of a stalk region with a conserved cysteine loop, a transmembrane domain, and a short cytoplasmic sequence ending with a postsynaptic density protein (PSD95), drosophila disc large tumor suppressor (DlgA), and zonula occludens‐1 protein (zo‐1) (PDZ)‐binding motif (Tabuchi and Südhof 2002). Mammalian Nxns are expressed from different promoters as the longer α and shorter β versions (Ushkaryov et al. 1992; Ushkaryovso et al. 1994). Regarding the trans‐synaptic complex, Nxns need to contain the alternative splice site 4 (+SS4) to bind Cblns (Cheng et al. 2016; Joo et al. 2011; Matsuda and Yuzaki 2011). Part of the complement component 1q (C1q)‐tumor necrosis factor superfamily, Cblns 1‐4 display spatially distinct expression throughout the brain (Miura et al. 2006; Seigneur and Südhof 2017). Their structure consists of a C1q‐like C‐terminal and a cysteine‐rich N‐terminal (Südhof 2023). For Cbln1, Cbln2, and Cbln4, the C‐terminal forms trimers, which are dimerized by disulfide bonds in the N‐terminal to assemble into homohexamers (Bao et al. 2006; Lee et al. 2012; Uemura et al. 2010). Meanwhile, Cbln3 assembles into heterohexamers and requires Cbln1 to function (Pang et al. 2000). Cbln1 and 2 have been shown to bind GluDs (Hirai et al. 2005; Ibata et al. 2019; Matsuda et al. 2010; Uemura et al. 2010), whereas Cbln4 mainly binds to the netrin receptor deleted in colon cancer (DCC) and neogenin‐1 (Finci et al. 2015; Haddick et al. 2014; Liakath‐Ali et al. 2022; Wei et al. 2012; Zhong et al. 2017). Isothermal titration calorimetry analyses suggested that two Nxn molecules bind to one Cbln molecule and two Cbln hexamers bind one GluD molecule, such that the triad is arranged in a 4:2:1 manner (4 Nxn monomers to 2 Cbln hexamers to 1 GluD hexamer) (Lee et al. 2012). However, more recent calorimetry studies rigorously demonstrate that one Nxn molecule binds to one Cbln hexamer, thereby resulting in a 2 Nxn: 2 Cbln: 1 GluD complex (Cheng et al. 2016; Elegheert et al. 2016) that forms a physical connection across the synaptic cleft to regulate synapse formation, maintenance, and plasticity.

Recent studies have also explored whether the trans‐synaptic complex may facilitate ion channel activity in GluDs. Studies in GluD2 suggested that the dimer interface, key to desensitization in other iGluRs, could be stabilized through either the formation of a trans‐synaptic complex or via chemical crosslinking of the dimers (Carrillo et al. 2021). Upon binding of glycine or D‐serine to GluD2 when in its trans‐synaptic state, either recreated using HEK293 cell clusters or present in cerebellar Purkinje neurons, leads to ionic conductance (Carrillo et al. 2021). However, these results were not reproduced (Itoh et al. 2024). Results were also not replicated for an ATD‐deleted GluD2 mutant, which showed current response without the cell cluster in Carrillo et al., but not in Itoh et al. Lack of replication can partly be explained by differences in the preparation of HEK293 cells, transfection efficiency, and variability in other parameters, suggesting that additional studies are needed to resolve this issue. Consistent with this, recent cryo‐EM studies and reconstitution of GluD2 in lipid bilayers suggest that D‐serine and GABA can induce conformational changes and ionic conductance in GluD2 (Wang et al. 2025). Because most of these studies have only studied GluD2, it will be important to examine GluD1 function in these conditions. The following sections will now focus on the current knowledge of GluD1 expression and roles in circuits and implications in human brain diseases.

## 5 Expression and Roles of GluD1 in the Brain

### 5.1 GluD1 at Excitatory Synapses

GluD1 has high expression in extracerebellar regions, such as in the hippocampal formation (Hepp et al. 2015; Konno et al. 2014; Nakamoto et al. 2019). GluD1 mRNA is expressed in vesicular glutamate transporter 1 (vGluT1)‐positive neurons (Konno et al. 2014) in the stratum lacunosum moleculare of the hippocampus and the molecular layer of the dentate gyrus, where it is required for excitatory synapse formation and maintenance (Tao et al. 2018). This synaptogenic role may be mediated through GluD1’s interaction with Cbln1, which is highly expressed in the entorhinal cortex, a major source of glutamatergic projections to the hippocampus (Miura et al. 2006; Seigneur and Südhof 2017). Deletion of Cbln1 results in reduced levels of vGluT1 in both the stratum lacunosum moleculare and dentate gyrus of 6‐month‐old, but not 1‐ and 2‐month‐old KO mice, suggesting a delayed onset of synaptic impairment (Seigneur and Südhof 2018). It should also be noted that Cbln2, which is expressed in the glutamatergic thalamic reuniens nucleus projection to the hippocampus (Seigneur and Südhof 2017), may also interact with GluD1 to mediate synaptogenic effects. Tao et al. demonstrated that the Nxn1β(+SS4)‐Cbln2‐GluD1 complex regulates AMPAR and NMDAR EPSCs as well as dendritic spine density in the hippocampus. Deletion of either Nxn1β(+SS4) or Cbln2 abolished AMPAR EPSCs, demonstrating that both binding partners are necessary for GluD1 to regulate excitatory transmission (Tao et al. 2018). Taken together, these studies suggest that GluD1 may differentially regulate excitatory transmission in the hippocampus, depending on the type of Cblns with which it interacts.

**FIGURE 2:**
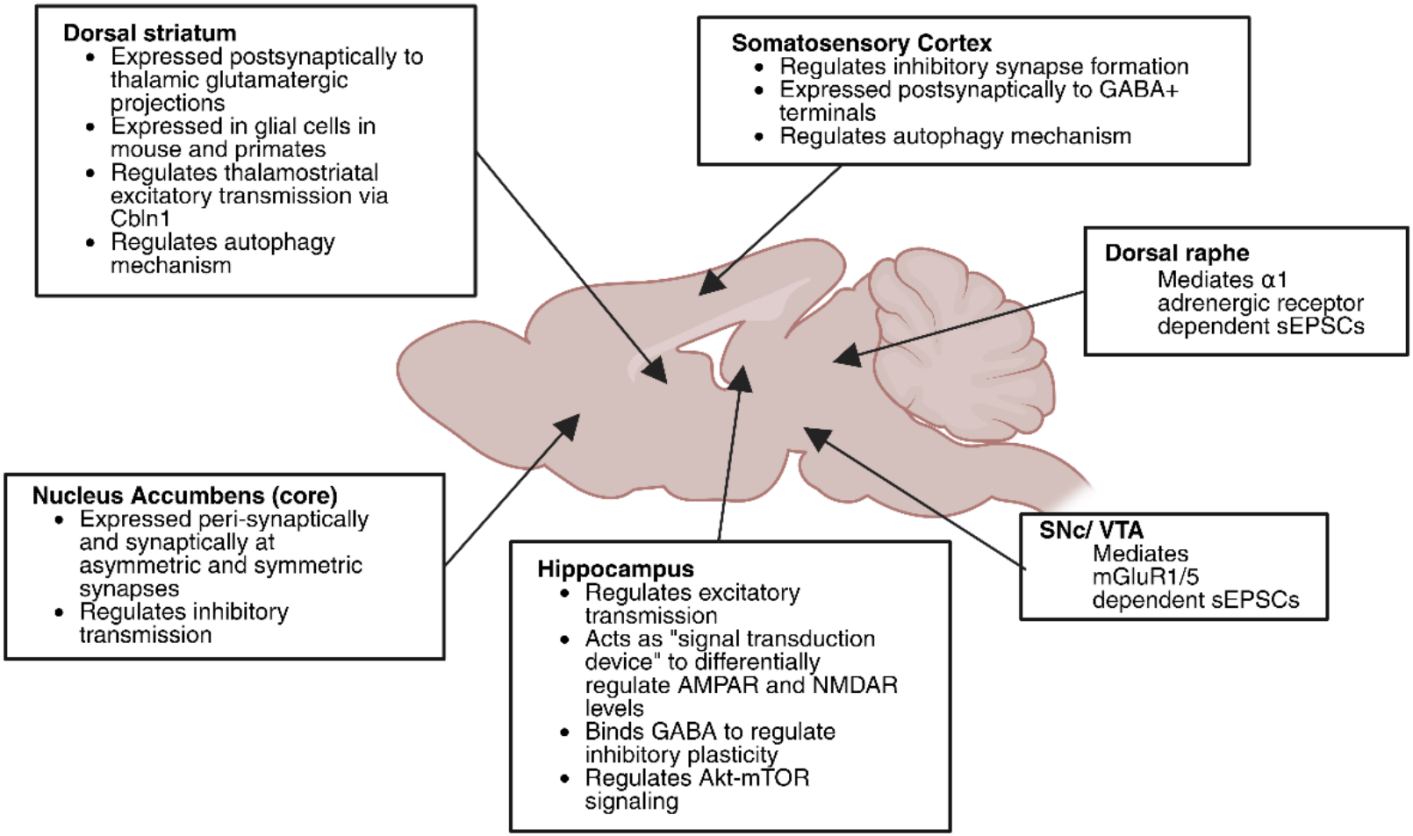
**GluD1’s various roles in the brain.** GluD1 plays various roles that encompass several brain regions, including the hippocampus, somatosensory cortex, dorsal striatum, midbrain, dorsal raphe, nucleus accumbens core and central amygdala. Discoveries are noted under each brain region.

Another region highly enriched in GluD1 is the dorsal striatum where it preferentially localizes at asymmetric axo‐dendritic synapses on medium spiny neurons (MSNs) (Hoover et al. 2021; Liu et al. 2020). Confocal imaging studies indicated that GluD1 colocalizes with vGluT2 over vGluT1 terminal‐like puncta (Liu et al. 2020). Knowing that excitatory thalamic and cortical projections to the striatum can be differentiated by vGluT2 and vGluT1, respectively (Fremeau et al. 2001), and that the parafascicular nucleus of the thalamus (Pf) is the main source of glutamatergic axo‐dendritic synapses onto striatal projection neurons (Dubé et al. 1998; Sadikot et al. 1992), these co‐localization data suggest that GluD1 preferentially associates with thalamostriatal over corticostriatal terminals. Upon conditional KO of GluD1 in the mouse dorsal striatum, Liu et al. demonstrated a significant reduction in miniature EPSC (mEPSC) frequency of MSNs as well as a reduction in vGluT2, but not vGluT1, puncta, and impaired behavioral flexibility. Selective ablation of GluD1 in the corticolimbic region did not affect behavioral flexibility, and no functional changes in excitatory transmission were observed at corticostriatal synapses upon striatal GluD1 deletion (Liu et al. 2020). Because Cbln1 is heavily expressed in the Pf (Kusnoor et al. 2010; Miura et al. 2006; Otsuka et al. 2016), it was suggested that GluD1 preferentially regulates excitatory thalamostriatal transmission at Pf‐MSN synapses in the striatum (Figure 2).

### 5.2 GluD1 at Inhibitory Synapses

Interestingly, GluD1 has also been shown to play a role in regulating inhibitory transmission (Fossati et al. 2019; Ryu et al. 2012; Yasumura et al. 2012). Elegant studies by Fossati et al. demonstrated that GluD1 regulates inhibitory synapses on cortical pyramidal neurons (Fossati et al. 2019). At the ultrastructural level, GluD1 is postsynaptically localized at synapses formed by vesicular γ‐aminobutyric acid (GABA) transporter (VGAT)‐positive terminals in juvenile cortical 2/3‐layer. Deletion of GluD1 via in utero electroporation resulted in a reduction of the frequency of miniature inhibitory postsynaptic currents (mIPSC) as well as a decrease in gephyrin cluster density in layer 2/3 cortical pyramidal neurons (Fossati et al. 2019). Mutating the NTD, CTD, or binding affinity for glycine or D‐serine in GluD1 also decreased the density of gephyrin clusters, indicating that GluD1 requires trans‐synaptic interactions to regulate the formation of inhibitory synapses in the cortex (Fossati et al. 2019). Fossati et al. further investigated the roles of Cblns 2 and 4, which are expressed in vasoactive intestinal peptide‐positive (VIP+) and somatostatin‐positive (SST+) interneurons, respectively (Paul et al. 2017; Tasic et al. 2016), in this phenomenon. It has previously been shown that Cbln4 knockdown in SST + cells decreased the density of synapses between SST + interneurons and cortical pyramidal neurons (Favuzzi et al. 2019). Similarly, knockdown of Cbln4, but not Cbln2, reduced the density of gephyrin clusters in juvenile layer 2/3 cortical pyramidal neurons (Fossati et al. 2019). Moreover, Cbln4 co‐immunoprecipitated with GluD1 and Cbln4 knockdown on GluD1 knockdown background did not further reduce the inhibitory puncta, suggesting they are part of the same signaling critical for inhibitory synapse formation (Fossati et al. 2019). However, as discussed previously, Cbln4 either binds weakly or not at all to GluDs (Matsuda and Yuzaki 2011; Rong et al. 2018; Wei et al. 2012), but instead interacts with DCC (Finci et al. 2015; Haddick et al. 2014; Wei et al. 2012) or neuogenin 1 (Liakath‐Ali et al. 2022). Thus, it remains unclear how Cbln4 and GluD1 may interact to facilitate inhibitory synapses. One possibility is that Cbln4 forms heteromers with other Cblns (Wei et al. 2012) that interact with GluD1 for the formation of inhibitory synapses. It is also possible that in the native system and specifically SST neurons, other mediators of inhibitory synapses facilitate interaction between GluD1 and Cbln4 homomers or Cbln1/4 heteromers. Finally, although Cbln4 is considered the only mediator of inhibitory synapses, it is possible that Cbln1 or Cbln2 may also participate in inhibitory synapses formation together with GluD1 given their ability to induce presynaptic differentiation of inhibitory synapses in in vitro studies (Yasumura et al. 2012).

Other studies also support GluD1’s role in regulating inhibitory synapses. For instance, GluD1 KO results in a decrease of hippocampal glutamate decarboxylase 67 (GAD67) levels (Yadav et al. 2012). Furthermore, in the nucleus accumbens core GluD1 is expressed peri‐synaptically and synaptically at both asymmetric (putatively glutamatergic) and symmetric (putatively GABAergic) synapses (Gawande et al. 2021). Loss of GluD1 in this region results in reduced mIPSC frequency and amplitude in MSNs as well as a reduction in the number of GAD67 puncta (Gawande et al. 2021). GluD1 also localizes to inhibitory synapses in the lateral habenula (LHb). In this region GluD1 was predominantly postsynaptic and frequently associated with GABA‐labelled symmetric synapses primarily on dendritic shafts in both rodents and primates, suggesting a conserved role for GluD1 at inhibitory synapses across species (Choi et al. 2025). The LHb receives major inputs from the entopeduncular nucleus and lateral hypothalamus and GluD1 is localized postsynaptically at both projections. Interestingly, GluD1 was found to be located in the core of symmetric synapses, but at the edges of asymmetric synapses. Similar localization of GluD1 on the edges of asymmetric synapses has been previously reported in the cortex (Nakamoto et al. 2019). As mentioned previously, GABA was found to be a low affinity ligand for GluD1 (Piot et al. 2023) and although GABA binding does not lead to ionic conductance in GluD1, it was found to be important for plasticity of inhibitory synapses. High frequency stimulation induces GluD1‐dependent long‐term potentiation of IPSC responses in the stratum lacunosum moleculare of the mouse hippocampus and this effect required GABA binding to GluD1 (Piot et al. 2023). D‐serine application also potentiated IPSCs in a GluD1‐dependent manner. Overall, these studies demonstrate both a structural and functional role of GluD1 in the formation, function and plasticity of inhibitory synapses in multiple brain regions.

### 5.3 GluD1 Regulation of Postsynaptic Properties and Intracellular Signaling

Additional studies have also proposed that GluD1 may have roles beyond strictly synaptogenesis. Dai et al. coined GluD1 as a “signal transduction device” that transduces extracellular Nxn‐Cbln signals to differentially regulate postsynaptic AMPAR and NMDAR activity without affecting synapse numbers (Dai et al. 2021). At hippocampal CA1‐subiculum synapses, the GluD1‐Cbln2 complex interacts with Nxn1(+SS4) and Nxn3(+SS4) to upregulate NMDAR and downregulate AMPAR EPSCs, respectively. This mechanism occurs with a minimal GluD1 construct, consisting of the Cbln2‐binding NTD, transmembrane region CD4, and C‐terminal cytoplasmic sequences containing motifs 3 and 4 (Dai et al. 2021), suggesting that transmembrane conformation change is not necessary to elicit these effects. Similar studies have evaluated Nxn1(+SS4)/Nxn3(+SS4) interactions with GluD1/2‐Cbln1/2 at other neural circuits (Dai et al. 2022; Südhof 2023). In the medial prefrontal cortex, Nxn1(+SS4) was shown to upregulate NMDAR activity via Cbln2, while in the cerebellum, Nxn3(+SS4)‐Cbln1‐GluD2 signaling downregulates AMPAR activity (Dai et al. 2022). Thus, GluDs can be differentially involved in the trans‐synaptic regulation of AMPAR and NMDAR in different brain regions.

Another interesting nonsynaptogenic role of GluD1 is its interaction with mGluRs. Functional coupling of GluDs and GαqPCRs (namely mGluR1/5 and α_1_‐adrenergic receptors) has been previously described. However, there is also evidence of GluD1/mGluR‐mediated regulation of downstream signaling pathways. At the subcellular level, GluD1 and group I mGluRs share similar patterns of localization, often found peri‐synaptically to asymmetric synapses (Gawande et al. 2021; Hepp et al. 2015; Luján et al. 1996; Masilamoni and Smith 2019; Mitrano and Smith 2006; Seigneur and Südhof 2018; Suryavanshi et al. 2016). This shared expression pattern suggests protein interactions between the two receptors. The mGluR5 is known to interact with the long‐form isomer of Homer to regulate downstream protein kinase B (Akt)—mammalian target of rapamycin (mTOR) signaling, protein synthesis, and LTD (Mao et al. 2005; Ronesi and Huber 2008). GluD1 deletion lowers mGluR5‐Homer interaction, leading to an increase in Akt and mTOR phosphorylation levels (Suryavanshi et al. 2016). Hyperactivity of the Akt–mTOR signaling pathway results in an increase in cell surface expression of AMPAR subunit 1 (GluA1) and GluA2, indicating impaired AMPAR endocytosis (Gawande et al. 2022; Suryavanshi et al. 2016). A similar relationship between GluD1 and mGluR5 has been reported in the somatosensory cortex, in which conditional GluD1 KO in excitatory corticolimbic neurons leads to an increase in phosphorylated Akt and mTOR levels. In these neurons, the hyperactivation of Akt–mTOR signaling impairs downstream autophagy mechanisms, as demonstrated by altered levels of autophagy proteins (i.e., sequestosome 1 (SQSTM1 or p62), beclin‐1, and the conversion of microtubule‐associated protein light chain 3 (LC3)‐1 to LC3‐II). The same study also described similar changes of dysregulated Akt–mTOR signaling and autophagy proteins in the dorsal striatum (Gawande et al. 2022). Taken together, these findings indicate that GluD1 is not simply a synaptogenic protein or a context‐dependent ion channel but a versatile signaling platform. Once integrated into trans‐synaptic complexes, GluD1 regulates AMPA and NMDA receptor incorporation at excitatory synapses through C‐terminal signaling. In addition, depending on the ligand engaged, GluD1 can independently drive depression at inhibitory synapses, highlighting its dual role in regulating both excitatory and inhibitory transmission. Similarly, GluD1 may contribute to tonic currents either independently or through interactions with the GPCRs, which regulate neuronal excitability. Framing GluD1 in this way integrates its neuronal functions into a unified model and provides a foundation for understanding how additional mechanisms may intersect with this signaling hub.

Extending this framework beyond neurons, early ultrastructural studies revealed that GluD1 is also expressed in glial compartments. In the striatum and LHb of both rodents and primates, GluD1 immunoreactivity has been detected in astrocytic processes adjacent to synapses, suggesting a potential role in glia (Choi et al. 2025; Hoover et al. 2021). Building on this anatomical evidence, it was demonstrated that GluD1 is also expressed in oligodendrocyte precursor cells (OPCs) and regulates OPC differentiation and myelination under both normal and demyelinating conditions (Gakare et al. 2023). A RNAseq analysis, has, indeed, shown that GluD1 is enriched in OPCs (Saunders et al. 2018). The potential functions of GluD1 in OPCs were evaluated using the GluD1 KO model. In normal conditions, deletion of GluD1 facilitated OPC differentiation and increased myelin basic protein levels (Gakare et al. 2023). In contrast, under demyelinating conditions, loss of GluD1 prevented recovery of myelin and produced more robust motor deficits. Neuron‐OPC synapses have been shown to contribute to proliferation, differentiation, and myelination, and it remains to be examined whether GluD1 may contribute to the formation of neuron‐OPC synapses, which may control these processes. A role of GluD1 in myelination is also relevant to neuropsychiatric disorders particularly schizophrenia where OPC and myelination dysfunction has been observed (Mauney et al. 2015; Yu et al. 2022). Additional studies are needed to more critically assess the role of GluD1 in glia and its potential implications for schizophrenia and demyelinating diseases.

## 6 GluD1 in Brain Diseases

### 6.1 Neuropsychiatric Disorders

**FIGURE 3:**
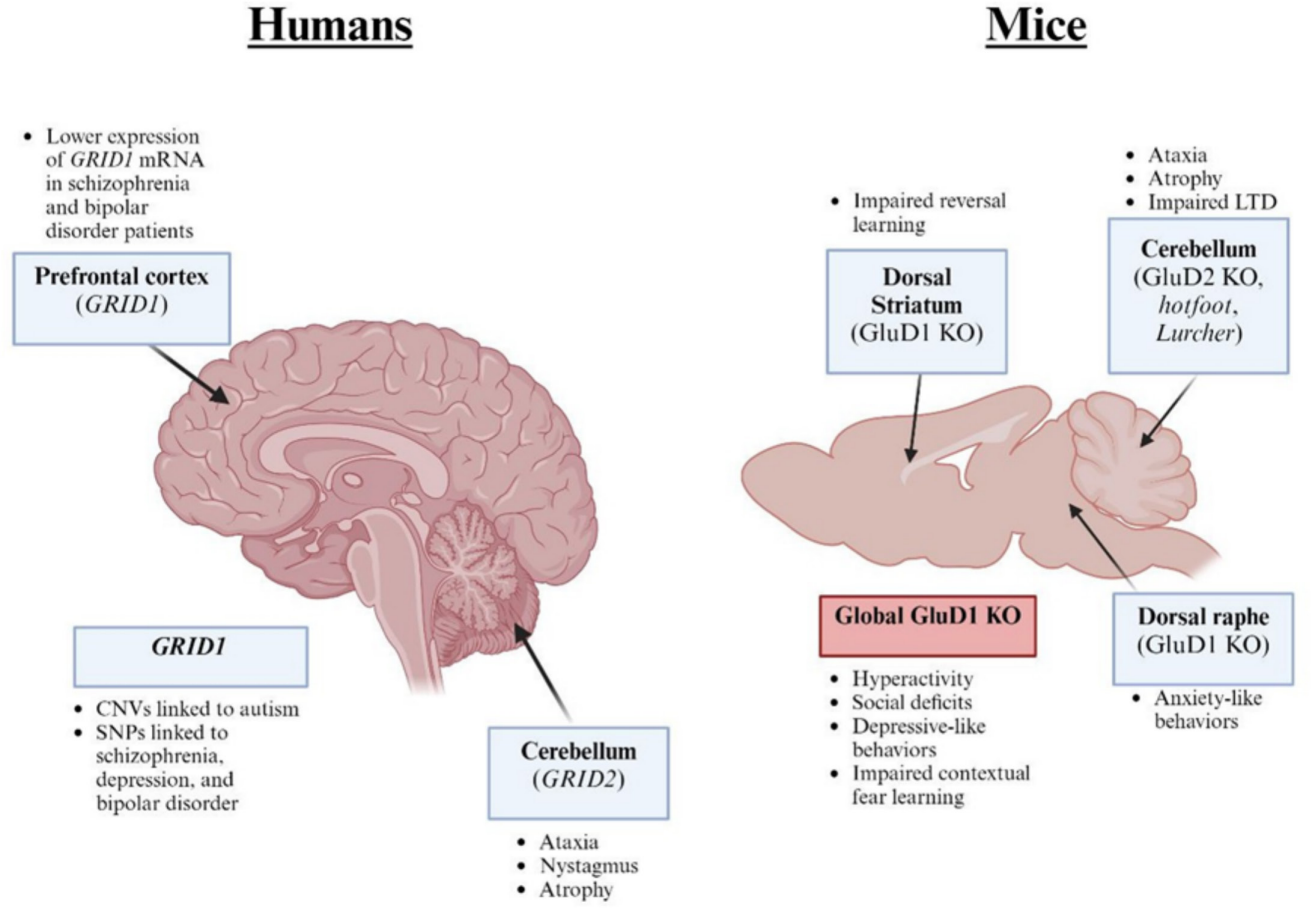
**GluDs in brain diseases.** GWAS have linked GRID1 and GRID2, the genes that encode for GluD1 and GluD2, respectively, with several neuronal disorders (left). Humans with GRID2 deletions exhibit a common phenotype that includes ataxia, nystagmus (or abnormal eye movements), and cerebellar atrophy. Copy number variations (CNVs) and single nucleotide polymorphisms (SNPs) in GRID1 are also linked to neuropsychiatric disorders, such as schizophrenia, depression, and bipolar disorder. Interestingly, global GluD2 and GluD1 KO mice distinct behavioral phenotypes, each mirroring human conditions linked to their respective expression patterns (right). GluD2 KO, as well as the hotfoot and Lurcher mutants, display motor ataxia, cerebellar atrophy, and impaired LTD. Global GluD1 KO mice exhibit hyperactivity, social deficits, and depressive‐like behaviors. GluD1 deletion in specific brain regions also gives rise to phenotypes that are consistent with the associated conditions in human patients.

As described above, GluD1 is highly enriched in brain regions implicated in the pathophysiology of neuropsychiatric disorders, including the dorsal striatum, which governs behavioral flexibility (Liu et al. 2020); the LHb (Choi et al. 2025), involved in reward processing, stress and depressive states; the bed nucleus of the stria terminalis (BNST) (Conley et al. 2025; Gandhi et al. 2021) and central amygdala (CeA) (Gandhi et al. 2021), which regulate emotion and anxiety, and the dorsal raphe (Gantz et al. 2020; Khamma et al. 2022), a key hub for serotonergic tone and mood regulation. In agreement with this, genome‐wide association studies have consistently identified GRID1, the gene encoding GluD1, as a susceptibility locus for several psychiatric conditions, including schizophrenia, autism spectrum disorder (ASD), major depressive disorder, bipolar disorder, and obsessive‐compulsive disorder (OCD) (Glessner et al. 2009; Griswold et al. 2012; Guo et al. 2007; Treutlein et al. 2009) (Figure 3). Supporting this, postmortem studies have reported reduced GRID1 mRNA expression in the prefrontal cortex of individuals with schizophrenia and bipolar disorder (Zhu et al. 2009).

Further evidence comes from the identification of rare pathogenic GRID1 mutations, such as p.Arg161His and p.Thr752Met, found in individuals with intellectual disability and spastic paraplegia. These variants impair mGlu1/5‐mediated calcium signaling and ERK activation and also lead to reduced dendritic complexity and excitatory synapse density (Ung et al. 2024). Broader analyses have identified additional missense variants clustered in functionally intolerant regions, including the ATD that mediates GluD1’s synaptogenic interactions with Cblns. The R341Q variant, for example, disrupts GluD1–Cbln2 binding while preserving surface expression, resulting in impaired synapse formation (Allen et al. 2024). Clinically, this mutation is associated with intellectual disability, ADHD, aggression, anxiety, and schizoaffective disorder. Another variant, A650T (Allen et al. 2024), found in schizophrenia, renders GluD1 constitutively active, mimicking the lurcher mutation.

Functional studies in GluD1 KO mice further link GluD1 to behavioral phenotypes relevant to neuropsychiatric disease. Deletion of GluD1 in the dorsal striatum impairs reversal learning and increases perseverative responses, modeling deficits in cognitive flexibility (Liu et al. 2020). In the NAc, GluD1 deletion leads to hypolocomotion, anxiety, depression‐like behavior (Gawande et al. 2021), and reduced behavioral sensitivity to psychostimulants by interfering with cocaine‐induced synaptic adaptations (Liu et al. 2018). These findings highlight distinct region‐specific roles of GluD1, with the dorsal striatum governing cognitive flexibility and the ventral striatum regulating affective and motivational states. Additionally, GluD1 KO mice show social impairment, including reduced sociability and poor social novelty recognition (Nakamoto et al. 2020; Yadav et al. 2012). These social behavior effects may arise due to GluD1–Cbln1 signaling at the VTA‐NAc pathway. Specifically, Ube3a‐overexpressing autism models show reduced Cbln1 in the VTA, leading to social deficits that are reversible upon Cbln1 restoration (Krishnan et al. 2017). Some of these impairments can be partially rescued by pharmacological interventions such as D‐cycloserine or selective serotonin reuptake inhibitors (Nakamoto et al. 2020; Yadav et al. 2012), highlighting a mechanistic link between GluD1 and neurotransmitter systems involved in social and affective function.

Anatomically, GluD1 is enriched in dendritic compartments of the LHb, where immunoelectron microscopy studies have localized it to GABAergic synapses from the lateral hypothalamus and entopeduncular nucleus (Choi et al. 2025). Given the LHb's central role in reward prediction error and stress sensitivity, and its hyperactivity in depression and schizophrenia, disrupted GluD1 signaling in this region may contribute to affective pathology via impaired inhibitory modulation. Mechanistically, GluD1 also participates in dopaminergic circuit regulation by forming a functional complex with mGlu1 in VTA neurons, where it drives slow depolarization required for burst firing, a key mode of signaling for reward prediction and salience detection. GluD1 loss abolishes burst firing, underscoring its necessity in dopaminergic function (Benamer et al. 2018). Beyond dopamine, GluD1 sustains tonic excitability of serotonergic neurons in the dorsal raphe nucleus, and its deletion produces anxiogenic behaviors (Copeland et al. 2023). In the BNST, GluD1 mediates tonic excitatory drive, further implicating it in stress and anxiety regulation (Conley et al. 2025; Gandhi et al. 2021). Collectively, these findings position GluD1 as a central synaptic organizer whose dysfunction, whether through genetic mutation, altered expression, or circuit‐level disruption, may contribute to neuropsychiatric disease pathology.

### 6.2 Neurological Disorders

Although GluD1 has been more widely studied in the context of neuropsychiatric conditions, emerging evidence indicates its relevance to a range of neurological disorders, including chronic pain (Gandhi et al. 2021; Sabnis et al. 2024, 2025; Shelkar et al. 2025), addiction (Liu et al. 2018), and motor dysfunction (Liu et al. 2020; Yadav et al. 2012, 2013). These disorders often involve maladaptive synaptic plasticity, altered circuit connectivity, and dysregulation of neurotransmitter systems, which can be attributed to GluD1 function.

**FIGURE 4:**
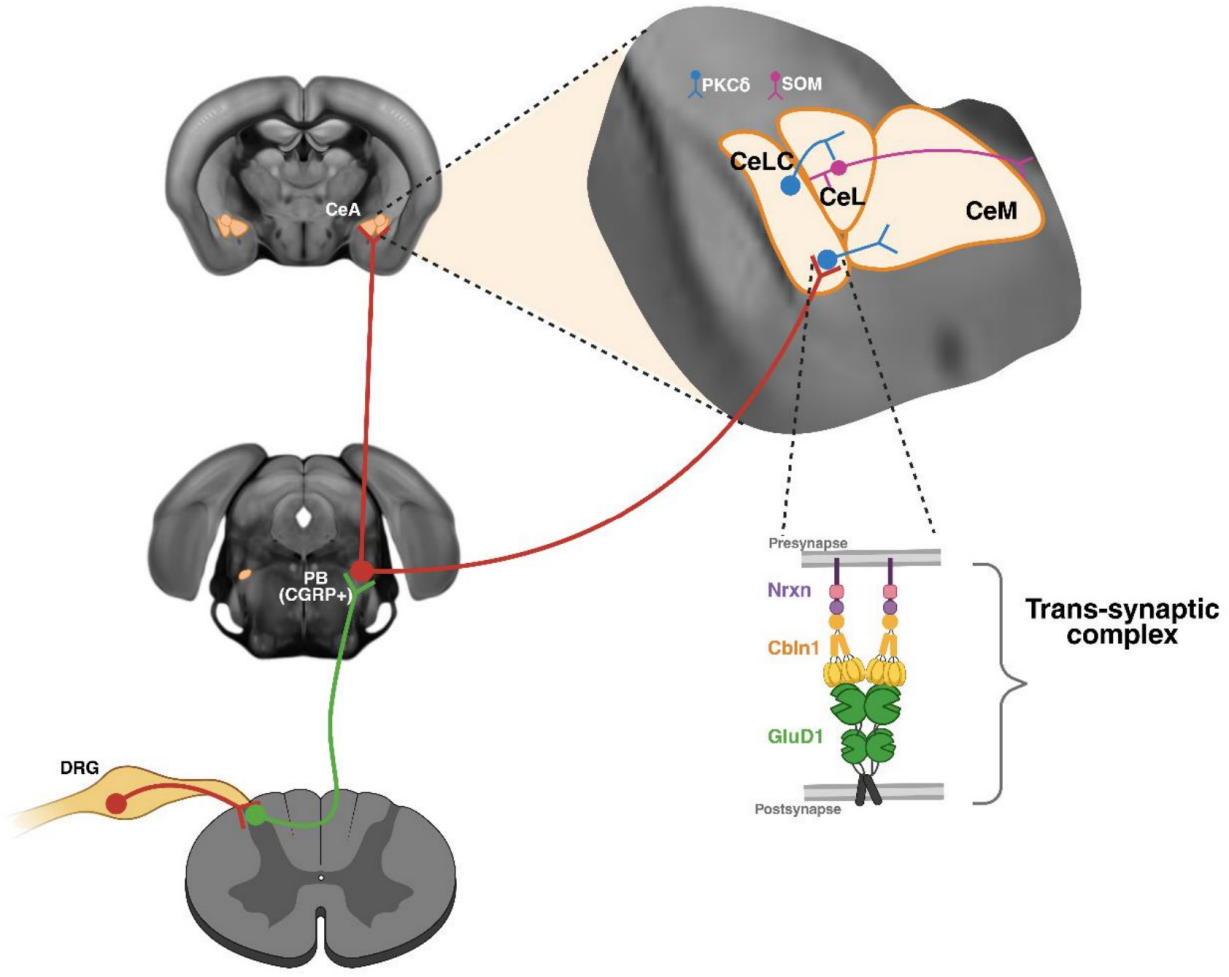
**The spino‐parabrachio‐amygdaloid pain pathway.** In the spino‐parabrachio‐ amygdaloid pain pathway, Lamina I neurons project to the external lateral PB, which, in turn, projects to the lateral capsular subdivisions of the CeA (CeLC). The PB is the exclusive source of CGRP to the CeLC and targets PKCδ(+) neurons (left). In the CeLC, GluD1 preferentially localizes in PKCδ(+) neurons, while Cbln1 is heavily expressed in PB/CGRP(+) terminals. Evidence suggests the GluD1‐Cbln1 complex regulates PB‐CeLC synapses to modulate pain behaviors in the mouse CeLC (right).

GluD1 is a crucial organizer of synaptic function within the CeA, particularly in the lateral capsular division (CeLC), a key region for the emotional‐affective dimension of pain. As indicated above, GluD1 localizes at calcitonin gene‐related peptide (CGRP)‐positive synapses from parabrachial (PB) neurons onto PKCδ+ pronociceptive neurons, forming a circuit critical for processing nociceptive input (Gandhi et al. 2021) (Figure 4). GluD1 is required for maintaining synaptic integrity in the CeA during chronic pain states. Disruption of GluD1–Cbln1 signaling leads to an increase in AMPA receptor expression in PKCδ+ neurons, potentially contributing to synaptic hyperexcitability and enhanced nocifensive behaviors in models of inflammatory and neuropathic pain (Gandhi et al. 2021; Shelkar et al. 2025). Recently, it was found that a GluD1‐mediated autophagy mechanism may underlie the neuroplasticity observed in the CeA in chronic pain (Narasimhan et al. 2025). Importantly, both intracerebroventricular and intravenous administration of recombinant Cbln1 restore GluD1 levels and alleviate mechanical hypersensitivity, supporting its translational potential (Gandhi et al. 2021; Sabnis et al. 2024). Additionally, in models of inflammatory pain, GluD1 downregulation occurs not only in the amygdala, but also along ascending nociceptive pathways, including the spinal cord, where recombinant Cbln1 treatment restores GluD1 expression and alleviates pain‐related behavior (Sabnis et al. 2024). Collectively, these findings position GluD1–Cbln1 signaling as a critical molecular hub in chronic pain, modulating CeA synaptic transmission, affective pain processing, and spinal nociceptive integration, and open the door to targeted interventions that address the cognitive and emotional dimensions of persistent pain.

GluD1 also contributes to motor learning and coordination via its role in dorsal striatal circuits (Liu et al. 2020). Conditional deletion of GluD1 from the dorsal striatum increases locomotor activity and latency to fall in rotarod test and affects thalamostriatal synaptic function (Liu et al. 2020). Although direct links to motor disorders remain to be established, these findings suggest that GluD1 dysfunction in basal ganglia pathways may be relevant to conditions such as Parkinson's disease, Tourette syndrome, or poststroke motor impairments, where striatal plasticity and input integration are compromised (Bradfield and Balleine 2017; Smith et al. 2014). Together, these studies expand the role of GluD1 beyond classical psychiatric circuits, demonstrating its function as a circuit‐specific modulator of synaptic plasticity in pain, reward, and motor networks. Its contribution to emotional‐affective pain signaling, experience‐dependent behavioral plasticity, and motor function underscores its potential as a therapeutic target across a broader spectrum of neurological conditions.

## 7 Conclusion

Our knowledge of GluD1 in brain functions is lacking substantially compared to other glutamate receptors. As more information pours in, it appears that GluD1 is capable of many unique features including its ability to bind multiple classes of neurotransmitters, supporting both excitatory and inhibitory synapse formation and modulation of synaptic plasticity and disease. Importantly, GluD1 and GluD2 also differ among each other, not only at the level of brain distribution, but also in structure–function, suggesting that any extrapolation from GluD2 studies to GluD1 needs caution. It is likely that many unknown features of GluD1 are yet to be discovered, which may have important implications on brain function and diseases.

## Author Contributions

**Diane Choi:** conceptualization, writing – original draft. **Poojashree B. Chettiar:** conceptualization, writing – original draft. **Yoland Smith:** conceptualization, writing – review and editing. **Shashank M. Dravid:** conceptualization, writing – original draft.

## Funding

This work was supported by National Institutes of Health (GM 8602‐24), National Institute of Mental Health (MH116003), National Institute of Neurological Disorders and Stroke (NS118731, NS132590, NS133338), and Office of Research Infrastructure Programs (OD011132).

## Conflicts of Interest

The authors declare no conflicts of interest.

## Data Availability

This review article does not contain any primary data.
